# Does salt stress affect the interspecific interaction between regionally dominant *Suaeda salsa* and *Scirpus planiculumis*?

**DOI:** 10.1371/journal.pone.0177497

**Published:** 2017-05-26

**Authors:** Jian Zhou, Lijuan Cui, Xu Pan, Wei Li, Manyin Zhang, Xiaoming Kang

**Affiliations:** 1 Institute of Wetland Research, Chinese Academic of Forestry, Beijing, China; 2 Beijing Key Laboratory of Wetland Ecological Function and Restoration, Beijing, China; 3 Beijing Hanshiqiao National Wetland Ecosystem Research Station, Beijing, China; Estacion Experimental del Zaidin, SPAIN

## Abstract

Plant-plant interactions that change along environmental gradients can be affected by different combinations of environmental characteristics, such as the species and planting density ratios. *Suaeda salsa* and *Scirpus planiculumis* are regionally dominant species in the Shuangtai estuarine wetland. Compared with non-clonal *S*. *salsa*, clonal *S*. *planiculumis* has competitive advantages because of its morphological plasticity. However, salt-tolerant *S*. *salsa* may grow faster than *S*. *planiculumis* in saline-alkali estuary soil. Whether the interactions between these two species along salinity gradients are affected by the level of salt stress and mixed planting density ratio remains unclear. Thus, to test the effects of salt stress and planting density ratios on the interactions between *S*. *planiculumis* and *S*. *salsa* in the late growing season, we conducted a greenhouse experiment consisting of 3 salinity levels (0, 8 and 15ppt) and 5 planting density ratios. Our results showed that the promotion of *S*. *salsa* growth and inhibition of *S*. *planiculumis* growth at low salinity levels (8 ppt) did not alter the interactions between the two species. Facilitation of *S*. *salsa* occurred at high salinity levels, and the magnitude of this net outcome decreased with increases in the proportion of *S*. *salsa*. These results suggest that competition and facilitation processes not only depend on the combinations of different life-history characteristics of species but also on the planting density ratio. These findings may contribute to the understanding of the responses of estuarine wetland plant-plant interactions to human modifications of estuarine salinity.

## Introduction

Plant-plant interactions play an important role in determining population dynamics and structuring ecological communities [1–2]. In recent decades, numerous studies have focused on plant-plant interactions along environmental gradients [3–5], and researchers have postulated the stress-gradient hypothesis (SGH). This hypothesis predicts that facilitation and competition have simultaneous effects on neighbouring individual plants, and the net outcome of this interaction will shift from negative to positive with increasing environmental stress [6–7]. Although this is a well-supported hypothesis, it is still debated with respect to specific issues, such as species traits and stress types [7–11].

Certain scientists oppose the SGH because of the inconsistent results observed between different studies [5, 12]. Maestre et al. [13] extended the SGH and proposed that the interactions between a single competitive pair will frequently transition from facilitation to competition along abiotic stress gradients (i.e., gradients of water, nutrient and environmental stress). In addition, effects are not only influenced by the physiological characteristics, functional traits and stress tolerance of the species but also by the combinations of different life-history characteristics of the species being tested. Previous studies found that the trade-offs between competitive ability and stress tolerance are key factors driving zonation patterns along environmental gradients [14–15]. Clonal plants with strong spatial expansion capabilities can fully exploit heterogeneous resources and avoid abiotic stress via their morphological plasticity in natural habitats [16–18]. To our knowledge, however, there is little experimental evidence on the interactions between clonal plants and stress-tolerant species transitioning from facilitation to competition along non-resource stress gradients.

Most tests of the SGH have focused on interactions between a single pair or a few pairs of species at a planting density ratio of 1:1 [19–22]. However, the individual plants were grown with different numbers of neighbouring species in natural habitats, and a species growing in a stressful environment may have a neighbour that exhibits different interaction strengths that may partly depend on the planting ratio. Interaction strength can be driven by the number of competitive neighbours as well as the environmental stress. However, few studies have measured the extent to which the proportions of species within the same habitat affect their interaction strength along environmental gradients [23].

Estuarine wetland plant communities are characterized by striking zonation patterns across salt stress gradients [14, 24–26]. Salinization is occurring at an unprecedented geographic scale that far exceeds natural variation trends, and these changes have profound consequences for wetland ecosystems [25, 27], especially estuaries. Thus, the mechanisms underlying variations in the relationship between salinity stress tolerance and competitive ability must be determined [28–30].

In the Shuangtai estuarine wetland, clonal *Scirpus planiculumis* and non-clonal *Suaeda salsa* are the dominant species in the saline-alkali field, and both species can coexist across broad ranges, from the low marsh to the terrestrial border of the marsh. Using such a combination of species to examine different responses to salt stress can reduce the confounding effects of other traits and reveal the importance of strategic traits on species interactions. To better understand the impact of salt stress and planting density ratio on the interactions between these two species, we addressed the following questions: (1) Do salt stress and the planting density ratio affect the growth of *S*. *planiculumis* and *S*. *salsa*? (2) Does the interspecific interaction between the two species along salinity gradients change depending on the planting density ratio? To answer these questions, we conducted a greenhouse experiment consisting of three levels of salt stress (low and high stress treatments and a no-stress control) and five planting density ratios (4:0, 3:1, 2:2, 1:3 and 0:4). Determining the competitive relationships between regionally dominant species will provide valuable insights for restoration practices for estuarine wetlands.

## Materials and methods

### Sampling sites

Plant samples were collected from the Shuangtai estuarine wetland, which is located at the north latitudes 40°45′ − 41°10′ and east longitudes 121°30′ − 122°00′ in Panjin, Liaoning Province of China. This wetland covers an area of 1579 km^2^ and experiences a semi-humid temperate monsoon climate, with a mean annual temperature of 8.4°C and mean annual precipitation ranging from 611.6 mm to 640.0 mm. In this ecosystem, the salinity values range from 0 to 34.7 ppt (parts per thousand, soil pore water salinity measured as micrograms NaCl per gram of water) in different areas, which affect the distribution and performance of halophytic plants [31–32].

### Species and propagation

The investigated plants were *S*. *salsa* (L.) and *S*. *planiculumis* Fr. Schmidt, which are dominant species in the Shuangtai estuarine wetland. *S*. *salsa* is an annual non-clonal halophyte in the family Chenopodiaceae, which is widely distributed along northern coasts consisting of saline alkali land. NaCl can accumulate in the vacuoles of this species to ensure plant survival under high salt stress [33]. *S*. *planiculumis* is a perennial, herbaceous clonal plant in the family Cyperaceae. In nature, the tubers of *S*. *planiculumis* can vegetatively produce new ramets to increase the plant’s ability to reproduce [34]. Both species commonly co-occur in the Shuangtai estuarine wetland.

The plants were collected from a single location in the wetland in the summer of 2015. Interactions between species are often affected by life-history stage, for instance, adult plants often compete as a consequence of facilitation of juvenile plants [11, 35]. To exclude temporal effects, we collected over 200 single adult plants, instead of juveniles, per species and grew them in a greenhouse for 10 days. This greenhouse located in the Wildlife Rescue & Rehabilitation Center, Beijing, China (the center is a protected area and specific permission should be issued by Beijing Municipal Bureau of Landscape and Forestry ahead of time). The plants were watered with tap water (no salt) to allow for acclimation to the indoor conditions before exposing them to the experimental salinity treatments. We discarded plants that exhibited any transplanting stress during these 10 days and then collected 150 single plants of *S*. *salsa* and 150 single ramets of S. *planiculumis*. We measured the average total dry mass and the average height of both species, and the initial respective measurements were 5.71 ± 0.93 g and 25.22 ± 5.22 cm for *S*. *salsa* and 3.24 ± 0.72 g and 38.99 ± 7.04 cm for *S*. *planiculumis*.

### Experimental design

Standard replacement series experiments have been widely used to evaluate interspecific interactions of mixed species, especially when studying interactions that involve only two species [36–37]. We grew *S*. *salsa* and *S*. *planiculumis* under three salt stress treatments: low salt stress (8 ppt), high salt stress (15 ppt) and a control treatment (no salt added). The salinity gradient was based on field measurements from related research [30, 38]. *S*. *salsa* and *S*. *planiculumis* were set up in mixtures and monocultures with five different planting density ratios: 0:4, 1:3, 2:2, 3:1 and 4:0. We set up 6 replicates for each treatment, and a total of 90 containers were used.

On 6 August 2015, according to the previously described cultivation density ratios, four plants of each species were transplanted in a vertical position into each experimental container. The containers were dark plastic barrels that measured 23 cm in diameter and 25.5 cm in depth, and they were filled with 12 cm of substrate, which was a 1:1 (v/v) mixture of sand and soil, which was obtained from the bank of an artificial lake in the Beijing Wildlife Rescue & Rehabilitation Center. The mixed substrate contained 0.31 (0.01) mg total N g^-1^ dry mass of soil (mean [SE]; N = 3), 0.55 (0.03) mg total P g^-1^, 1.33 (0.09) mg K g^-1^, and 8.66 (0.71) mg organic matter g^-1^ based on analysis conducted at the Institute of Botany at the Chinese Academy of Sciences in Beijing.

On 13 August 2015, which represented one week of establishment, we slowly added an NaCl solution to the containers. According to the volume of the substrate in a container, we added 40.57 g and 76.06 g sodium chloride (99.59% purity) into separate containers for the low and high salt treatments, respectively, and we prepared 1 L of sodium chloride solution and added this to each treatment container. We added 1 L of tap water to the control containers. The NaCl solution was only added at the beginning of the experiment. During the experiment, the containers were arranged in blocks and were watered twice a week to minimize growth limitations caused by water availability. During the experiment, the mean temperature in the greenhouse was 21.2°C and the relative humidity was 75.5%.

On 22 October 2015, we harvested the experiment. The 10-week experimental duration was sufficient for these two species to reach maturity and exhibit peak biomass because both species had blossomed before the end of the experiment, and all plants survived. We measured the heights of the plants, and then each plant was divided into above- and belowground parts, dried at 70°C for over 72 hours and weighed because biomass is an important growth index to measure interspecific competition.

### Data analysis

We analysed the growth of *S*. *salsa* and *S*. *planiculumis* and performed two-way ANOVAs to test the effects of the different levels of salt stress (no-salt control, 8 ppt low salt and 15 ppt high salt) and planting density ratios of these species (4:0, 3:1, 2:2, 1:3 and 0:4) on the total biomass, aboveground biomass, belowground biomass and average height. Differences were tested using Tukey’s *post hoc* honest significant difference test. In the ANOVA models, salt stress and the planting density ratio were treated as fixed effects. The mean values of all plants in a container were used in these analyses.

Based on the total biomass data of *S*. *salsa* and *S*. *planiculumis*, we analysed the competitive response using the relative interaction index (RII). The RII is suitable for calculating the positive and negative interactions between plants because this index can compare the performance of each species grown in a mixture to the performance of the species grown in a monoculture. In addition, the competition intensity between two species can effectively be measured because the RII often better meets the assumptions for statistical analysis compared with alternative competitive models [39–40]. The calculation formulae are as follows:

$$\begin{array}{l} RII_{a}=(Y_{ab}-Y_{a})/(Y_{ab}+Y_{a});\\ RII_{b}=(Y_{ba}-Y_{b})/(Y_{ba}+Y_{b}) \end{array}$$

where Y is the total biomass per plant in each experimental container; *a* and *b* represent the two species; Y_a_ is the total biomass of species *a* when grown alone; Y_b_ is the total biomass of species *b* when grown alone; Y_ab_ is the total biomass of species *a* when grown with species *b*; and Y_ba_ is the total biomass of species *b* when grown with species *a*. If the RII value is 0, there are no significant differences between the mixtures and the monoculture; if the RII value is positive; then the interaction is facilitative; and if the RII value is negative, the interaction is competitive. Significant deviations of RII values from zero at the *P* = 0.05 level were determined using a *t*-test. All analyses were conducted using SPSS 20.0 (Statistical Product and Services Solutions, version 20.0; SPSS Inc., Chicago, IL).

## Results

### Effects of salt stress and planting density ratio on the growth of the two species

**Fig 1:**
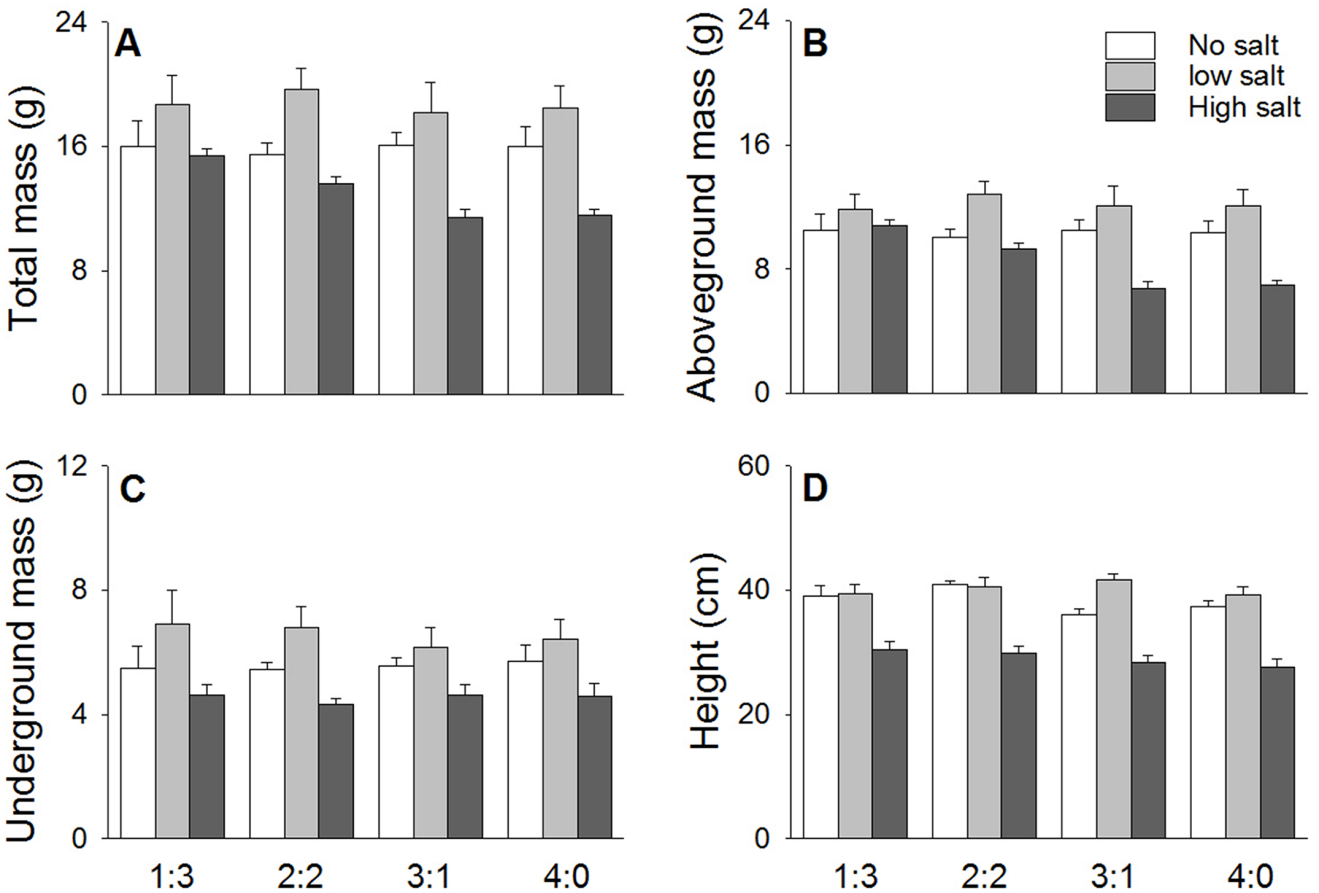
Effects of salt stress (no salt, low salt and high salt) and planting density ratio (proportions of *S*. *salsa* and *S*. *planiculumis* were 1:3, 2:2, 3:1 and 4:0) on the (A) total mass, (B) aboveground mass, (C) belowground mass and (D) height (mean + SE) of *S*. *salsa*.

As predicted, the biomass of *S*. *salsa* decreased significantly with increasing salt stress (*P* < 0.001, Table 1 and Fig 1). The low salt treatment resulted in the highest biomass value of *S*. *salsa*, which was approximately 15% higher than that in the control treatment and 40% higher than that in the high salt stress treatment. The treatments with different mixed planting density ratios did not affect the total biomass (*F*_3, 60_ = 1.06, *P* = 0.372) or height (*F*_3, 60_ = 2.08, *P* = 0.112) of *S*. *salsa* (Table 1).

**Table 1:** ANOVA results for the effects of salt stress and interspecific competition on the growth of *S*. *salsa* and *S*. *planiculumis*.

Response variables	Salt stress (S)		Competition (C)		S * C	
	*F*_2, 60_	*P*	*F*_3, 60_	*P*	*F*_6, 60_	*P*
***Suaeda salsa***						
Total mass	23.12	**< 0.001**	1.06	0.372	0.86	0.530
Aboveground mass	22.44	**< 0.001**	2.04	0.118	2.16	0.060
Belowground mass*	13.41	**< 0.001**	0.08	0.969	0.21	0.973
Height	94.58	**< 0.001**	2.08	0.112	1.34	0.252
***Scirpus planiculumis***						
Total mass	163.46	**< 0.001**	0.91	0.440	0.28	0.945
Aboveground mass	149.20	**< 0.001**	0.96	0.418	0.37	0.895
Belowground mass*	97.19	**< 0.001**	0.34	0.796	0.18	0.962
Height	55.79	**< 0.001**	3.17	**0.031**	1.24	0.297

*These data were transformed to meet the requirements for homoscedasticity and normality. Bold type indicates a significant difference (*P* < 0.05).

**Fig 2:**
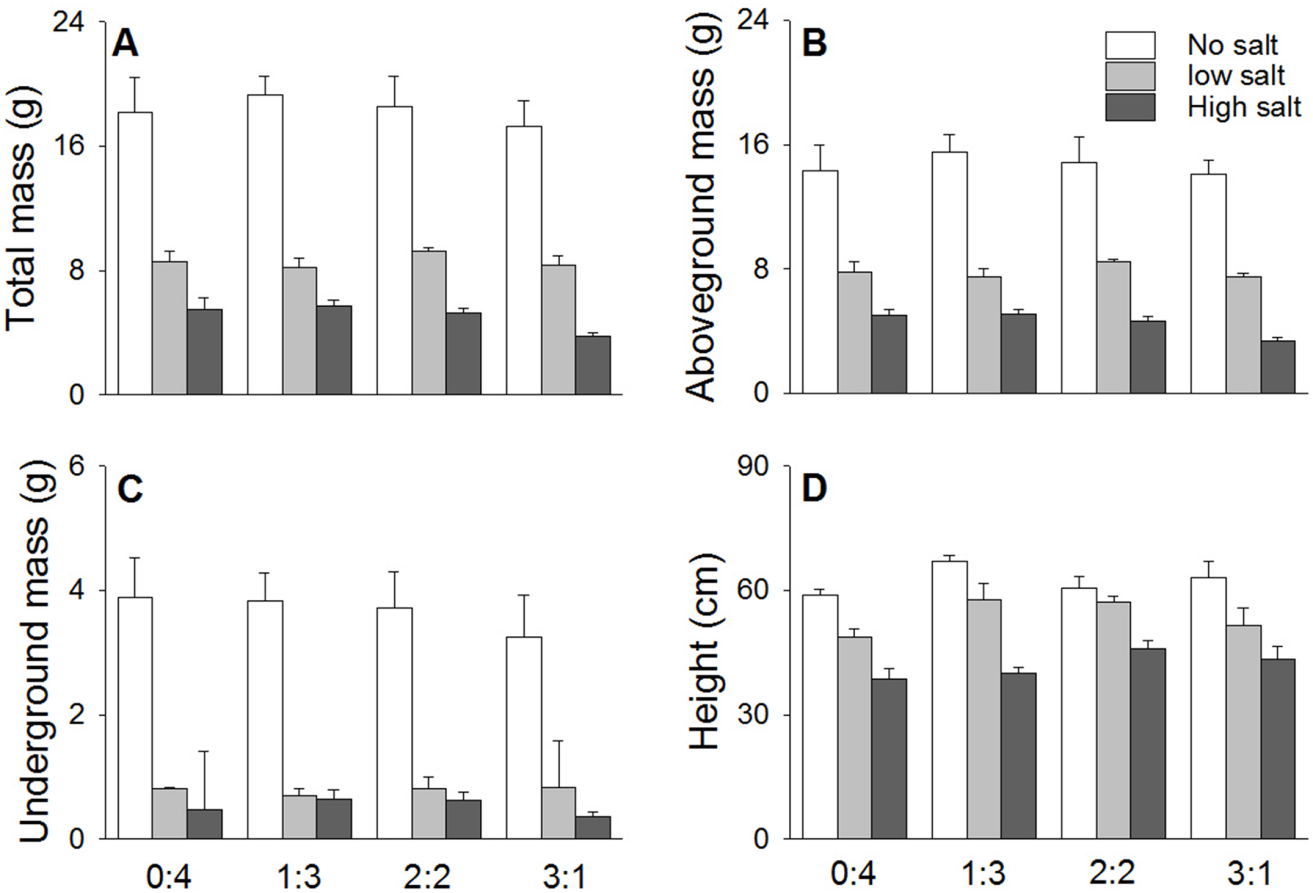
Effects of salt stress (no salt, low salt and high salt) and density ratio (proportions of *S*. *salsa* and *S*. *planiculumis* were 0:4, 1:3, 2:2 and 3:1) on the (A) total mass, (B) aboveground mass, (C) belowground mass and (D) height (mean + SE) of *S*. *planiculumis*.

Similar to the results for *S*. *salsa*, the salt stress treatments also had an obviously negative effect on the growth of *S*. *planiculumis*. The control treatment resulted in the highest biomass value of *S*. *planiculumis* (Table 1 and Fig 2), which was approximately 60% lower in the low salt treatment and 80% lower in the high salt treatment. Significant differences were not observed in the total biomass (*F*_3, 60_ = 0.91, *P* = 0.440), aboveground mass (*F*_3, 60_ = 0.96, *P* = 0.418) and belowground mass (*F*_3, 60_ = 0.34, *P* = 0.796) of *S*. *planiculumis* across the different planting density ratios, although the presence of *S*. *salsa* significantly enhanced the height of *S*. *planiculumis* (*F*_3, 60_ = 3.71, *P* = 0.031). No interactions were observed between the treatments.

### RII results

**Fig 3:**
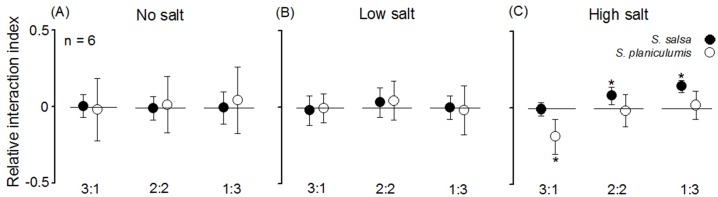
One sample t-test results for the differences between the RII values and zero. The results are for *S*. *salsa* and *S*. *planiculumis* under different density ratios (proportions of *S*. *salsa* and *S*. *planiculumis* were 3:1, 2:2 and 1:3) and salt treatments: (A) no salt, (B) low salt and (C) high salt. The mean values and 95% confidence intervals are shown, and * indicates a significant difference (*P* < 0.05).

In the no salt and low salt treatments, the RII values were close to zero for both *S*. *salsa and S*. *planiculumis* (*P* > 0.05, Table 2). In the high salt treatments, the RII values of *S*. *salsa* were significantly greater than zero when the proportions of *S*. *salsa* and *S*. *planiculumis* were 2:2 and 1:3 (Table 2; Fig 3), which indicates that the salt-tolerant species *S*. *salsa* were benefactor and clonal *S*. *planiculumis* were beneficiary in this case. The RII values of *S*. *planiculumis* were strongly negative when the planting density ratio was 3:1, indicating that the effects of *S*. *salsa* on *S*. *planiculumis* were competitive (Table 2; Fig 3).

**Table 2:** One sample *t*-test results for *S*. *salsa* and *S*. *planiculumis* under different density ratios and salt stress treatments. *P* values are shown.

Species	df	No salt			Low salt			High salt		
		3:1	2:2	1:3	3:1	2:2	1:3	3:1	2:2	1:3
*S*. *salsa*	5	0.83	0.86	0.63	0.88	0.42	0.77	**0.01**	0.67	0.66
*S*. *planiculumis*	5	0.88	0.72	0.87	0.62	0.42	0.96	0.60	**0.02**	**<0.01**

Bold type indicates a significant difference (*P* < 0.05).

## Discussion

Salt stress is one of the main environmental factors that affect plant growth and morphology in estuaries [24]. High salt concentrations (15 ppt) significantly decreased the growth of the two studied species, and the osmotic effect of the salt damaged the roots of both species [41], which subsequently inhibited the growth of the plants. Photosystem II in *S*. *salsa* showed high resistance to low salinity [42], which makes the plant more suitable for growth under low salinity environments (8 ppt) than *S*. *planiculumis*. Thus, *S*. *salsa* reached its maximum biomass in the low salt treatment. These results are consistent with previous studies on the effects of salt stress on plants.

Different planting density ratios of *S*. *salsa* and *S*. *planiculumis* did not significantly affect the biomasses of the two species in any of the salinity treatments. However, the presence of *S*. *salsa* greatly increased the average height of *S*. *planiculumis* (*P* = 0.031, Table 1), which may be related to the morphological plasticity of clonal plants such as *S*. *salsa*, which can absorb more light and take up more space under competition. Many studies have also shown that clonal reproduction appears to select for escape strategies when plants experience competition [43–44].

The RII results for *S*. *salsa* and *S*. *planiculumis* along the salinity stress gradient and under the different planting density ratios were complex. Both competitive and facilitative processes were asymmetrical for *S*. *salsa* and *S*. *planiculumis* across the salinity stress gradients. In fresh water and low salinity water across all density ratios, plants interactions tended to be less facilitative relative to the high stress treatments (Fig 3), these results support the SGH. One possible explanation for these results may be related to the intensity of interspecific interaction between these two species, which was equal to their intraspecific interaction under low abiotic stress conditions. Another explanation may be that the fresh water and low salinity stress gradients were too weak to constitute a major proportion of the species’ niches. In other words, these species were not sensitive to these two levels [7, 10]. Moreover, He et al. [15] found that the stress-tolerant and competitively inferior *S*. *salsa* did not benefit from *T*. *chinensis* amelioration of abiotic stress, which is partly consistent with our findings. The duration of the growing season may also be a reasonable explanation for the almost non-existent interaction under low abiotic stress conditions.

The balance between competition and facilitation was disturbed when the salinity was increased to 15 ppt according to the RII results (Fig 3C). *S*. *salsa* benefitted from the interaction under increased salt stress because of its high salt tolerance, especially when this species was present in the minority (*S*. *salsa*: *S*. *planiculumis* density ratio of 2:2 or 1:3). As predicted by Maestre et al. [13], plant-plant interactions will shift from competition to facilitation with increased abiotic stress for plant-plant combinations with dissimilar competitive abilities and stress tolerances, and this shift has been observed in previous studies [6, 22, 45–46]. However, the facilitative effects disappeared with increasing numbers of *S*. *salsa* because the intraspecific interactions between *S*. *salsa* plants inhibited their growth. This result suggests that the planting density ratio affects the interactions between the two species as they shift from competition to facilitation under high salinity.

## Conclusions

Our study demonstrated that (1) the growth of *S*. *planiculumis* was inhibited under both low and high salinity conditions, whereas the growth of *S*. *salsa* was only inhibited under high salinity. The planting density ratios did not alter the growth of either *S*. *planiculumis* or *S*. *salsa*. (2) The promotion of *S*. *salsa* growth and the inhibition of *S*. *planiculumis* growth at low salinity (8 ppt) did not alter their interactions. (3) Facilitation occurred at high salinity, and the magnitude of this net outcome decreased with increases in the proportion of *S*. *salsa*. These results provide insights into the organization and assembly of estuarine wetland plant communities and may have important implications for our understanding of the responses of estuarine wetland plant communities to human modifications of estuarine salinity. However, the results may depend on a variety of biotic and abiotic environmental stress factors and complex combinations of these factors in natural habitats. Further studies should examine shifts between competition and facilitation under multiple types of environmental stresses in estuarine wetland plant communities.
